# Impact of COVID-19 on health services utilization in Province-2 of Nepal: a qualitative study among community members and stakeholders

**DOI:** 10.1186/s12913-021-06176-y

**Published:** 2021-02-24

**Authors:** Devendra Raj Singh, Dev Ram Sunuwar, Sunil Kumar Shah, Kshitij Karki, Lalita Kumari Sah, Bipin Adhikari, Rajeeb Kumar Sah

**Affiliations:** 1 Department of Public Health, Asian College for Advance Studies, Purbanchal University, Satdobato, Lalitpur, Nepal; 2 Research and Innovation Section, Southeast Asia Development Actions Network (SADAN), Lalitpur, Nepal; 3 Research Section, Swadesh Development Foundation (SDF), Siraha, Province-2 Nepal; 4 Department of Nutrition and Dietetics, Armed Police Force Hospital, Kathmandu, Nepal; 5 Program Section, Bagmati Welfare Society Nepal, Sarlahi, Province-2 Nepal; 6 Faculty of Medicine, Health and Social Care, Canterbury Christ Church University, Kent, UK; 7 Mahidol-Oxford Tropical Medicine Research Unit, Faculty of Tropical Medicine, Mahidol University, Bangkok, Thailand; 8 Centre for Tropical Medicine and Global Health, Nuffield Department of Medicine, University of Oxford, Oxford, UK

**Keywords:** COVID-19, Health services, Health care utilization, Pandemic, Community experiences, Nepal

## Abstract

**Background:**

The COVID-19 pandemic has posed unprecedented challenges and threats to the health care system, particularly affecting the effective delivery of essential health services in resource-poor countries such as Nepal. This study aimed to explore community perceptions of COVID-19 and their experiences towards health services utilization during the pandemic in Province-2 of Nepal.

**Methods:**

The semi-structured qualitative interviews were conducted among purposively selected participants (*n* = 41) from a mix of rural and urban settings in all districts (*n* = 8) of the Province 2 of Nepal. Virtual interviews were conducted between July and August 2020 in local languages. The data were analyzed using thematic network analysis in NVivo 12 Pro.

**Results:**

The findings of this research are categorized into four global themes: i) Community and stakeholders’ perceptions towards COVID-19; ii) Impact of COVID-19 and lockdown on health services delivery; iii) Community perceptions and experiences of health services during COVID-19; and iv) COVID-19: testing, isolation, and quarantine services. Most participants shared their experience of being worried and anxious about COVID-19 and reported a lack of awareness, misinformation, and stigma as major factors contributing to the spread of COVID-19. Maternity services, immunization, and supply of essential medicine were found to be the most affected areas of health care delivery during the lockdown. Participants reported that the interruptions in health services were mostly due to the closure of health services at local health care facilities, limited affordability, and involvement of private health sectors during the pandemic, fears of COVID-19 transmission among health care workers and within health centers, and disruption of transportation services. In addition, the participants expressed frustrations on poor testing, isolation, and quarantine services related to COVID-19, and poor accountability from the government at all levels towards health services continuation/management during the COVID-19 pandemic.

**Conclusions:**

This study found that essential health services were severely affected during the COVID-19 pandemic in all districts of Province-2. It is critical to expand and continue the service coverage, and its quality (even more during pandemics), as well as increase public-private sector engagement to ensure the essential health services are available for the population.

**Supplementary Information:**

The online version contains supplementary material available at 10.1186/s12913-021-06176-y.

## Background

The Coronavirus infectious disease (COVID-19) pandemic has created tremendous challenges and threats to human life and health systems globally [1, 2]. In the absence of definitive curative measures for COVID-19, current infection control and management rely heavily on public health-preventative measures, including lockdown and social distancing, to prevent disease transmission [3]. By the end of March 2020, more than one hundred countries were in either partial or full lockdown to mitigate the spread of COVID-19 infection [4], thus compelling millions of people to remain housebound [5]. The lockdown measures have severely affected economic activities, population health and mental wellbeing, health care delivery, and health services utilization, particularly in resource-poor countries such as Nepal [6–8]. Nepal is considered as one of the highly vulnerable countries for the COVID-19 pandemic with minimum resources to tackle the COVID-19 outbreak [9, 10].

On 24 March 2020, the Government of Nepal imposed a nationwide lockdown which affected the day-to-day activities of the majority population in both rural and urban areas of Nepal. This lockdown came at a very early stage of the pandemic in Nepal and well before COVID-19 community transmissions were observed in the country [11]. The lockdown included the closure of transportation services, markets, city centers, and even the outpatient services in hospitals [6]. In addition, the majority of peripheral health facilities were closed and routine essential health services were disrupted for several months because of the unavailability of personal protective equipment (PPE) for health workers [6, 7]. Nepal has already been facing a high prevalence of maternal deaths, child deaths, different forms of malnutrition, and the burden of mortality and morbidity associated with both non-communicable and communicable diseases, which may have worsened further by the disruption of health services due to the lockdown [12].

The lockdown measures due to COVID-19 have jeopardized Nepal’s decade-long significant progress, especially in the areas of maternal and child health, for example, maternal mortality and neonatal deaths have suddenly increased by more than three folds during the pandemic [13]. Childbirth at home in the absence of skilled health care workers and the prevalence of morbidities associated with other communicable and non-communicable diseases have also significantly increased after the COVID-19 outbreak in Nepal [8, 13]. The COVID-19 pandemic is anticipated to further worsen the availability and utilization of health services in both urban and rural areas of Nepal. As of February 1, 2021, there have been 271,118 confirmed COVID-19 cases with 2029 deaths in Nepal [14]. This study was conducted in Province 2, which is the worst affected province of Nepal, with a concentration of nearly 50% of the total COVID-19 cases and related deaths [15].

### Health care structure in Nepal

After the promulgation of a new constitution in 2015, Nepal has undergone a radical transformation from a unitary administrative system to the three tiers of decentralized governments: a federal government, seven provincial governments, and 753 local governments [16]. In addition, the health care structure is also devolved into three tiers (federal, provincial, and local health systems) with more autonomy to the local governments. At the local or community level health facilities (municipality or ward level), urban health clinics, health posts, and primary health care centers are the primary contacts for health services [17, 18]. Female community health volunteers (FCHVs), supported by outreach clinics, urban health clinics, and health promoters are frontline health cadres at the community level [19, 20]. District hospitals are the secondary care referral health centers at the district level [20]. Hospitals under the provincial and federal governments are the tertiary care referral centers [17, 20]. Private health care facilities, which are mostly expensive and unaffordable available throughout the country mainly as polyclinics, pharmacies, nursing homes, and hospitals [21].

The Government of Nepal has been responding to tackle the COVID-19 pandemic through the implementation of two key strategic approaches: public health prevention and hospital-based interventions [22]. Despite the government’s efforts in mitigating the COVID-19 pandemic, the current crisis has severely affected the health care system of the country. However, little is known about how the COVID-19 crisis is affecting health services availability and utilization among the population in Nepal. To the best of our knowledge, this is the first qualitative study that aims to explore community perceptions and experiences towards health services utilization during the COVID-19 pandemic in Nepal. Community members in this study refer to all population, including health care workers, in the community regardless of their age, sex, education, occupation, or any other demographic characteristics. In this study, we have adopted the Andersen health care utilization model as a theoretical basis to explore the factors influencing community experiences in health services utilization in the context of COVID-19 in Nepal [23, 24]. The model includes environmental factors (health system factors and external factors), population characteristics (predisposing characteristics, enabling resources, and need factors), health behavior factors (personal health behaviors and utilization of health services), and outcome factors (community satisfaction towards health services utilization) [23].

## Methods

This study utilized qualitative research methodology to explore community perceptions towards COVID-19 and their experiences towards health services utilization during the COVID-19 pandemic in Province − 2 of Nepal. The development and reporting of this study followed the standard guidelines of consolidated criteria for reporting qualitative studies (COREQ) [25].

### Study setting

**Fig. 1:**
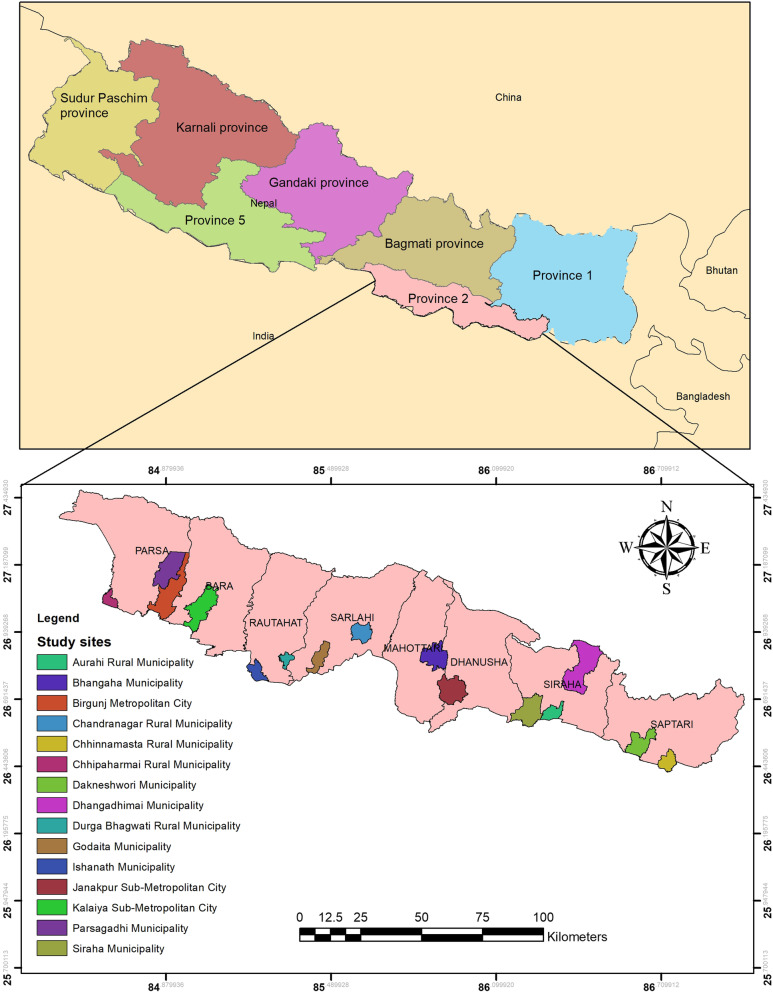
Map showing urban and rural study sites in eight districts in Province 2 of Nepal. The map was created using ArcGIS desktop version 10.8. The shape files of the administrative Province and districts for Nepal were obtained from the Government of Nepal, Ministry of Land Management, Survey Department website that were publicly available for unrestricted use (http://www.dos.gov.np/nepal-map)

This study was conducted in all eight districts of Province 2, located in Southern Nepal (Fig. 1). It is one of the seven self-governed provinces of Nepal, which mostly constitutes the Terai region (Southern plain land) with an area of 9661 km2 and is the second-most populous province of Nepal with a total population of 5,404,145 [16]. The majority of the population in the province comes from *Madheshi* ethnic groups and speak *Maithili*, *Bhojpuri*, *Bajjika*, *Nepali*, *Abhadhi*, and other local languages [16]. The eight districts of the province are further divided into one metropolitan city, three sub-metropolitan cities, 73 municipalities, and 59 rural municipalities [16]. According to the Nepal Demographic and Health Survey 2016, province 2 fares poorly in most health status indicators [26]. For example, the province has a high prevalence of teenage pregnancy (27%) [26], home delivery without a skilled birth attendant (55%), multidimensional poverty (48%) [27], and illiteracy (50%) [28]. Evidence suggests that health services utilization amongst pregnant women in this province is very low with only 26% of mothers utilizing maternal health services (combined antenatal care, delivery care, and postnatal care) [29]. The province also suffers from poor health care infrastructure with limited availability of specialized health care services [29]. This province is also considered as one of the most vulnerable regions for COVID-19 where all eight districts of the province share a porous border with India with a significant movement of labor migrants [15] and this is reflected with the highest number of COVID-19 cases compared to other provinces.

### Research participants

The study used purposive sampling [30] to select 41 participants from all 8 districts of Province 2. This sampling method allowed achieving maximum diversity of participants in terms of sex, age, education, occupation, and residence (Table 1). Community members and stakeholders (health care workers, female community health volunteers, local community representatives, teachers, social workers, and journalists) were selected based on their age (above 18 years), permanent resident of the Province-2, workplace at selected sites, and able to respond over a phone call for the interview.

**Table 1:** Characteristics of study participants

Participant No. (P#)	Sex	Age range (years)	Education	Occupation	Rural/Urban Municipality	District
P 1	F	46–50	Secondary level	FCHV	Urban	Saptari
P 2	F	21–25	Higher secondary level	Student	Urban	Saptari
P 3	M	36–40	Secondary level	Healthcare worker	Urban	Saptari
P 4	M	46–50	Higher secondary level	Ward Chairperson	Urban	Saptari
P 5	M	41–45	Secondary level	Health Coordinator	Rural	Saptari
P 6	F	21–25	Higher secondary level	Unemployed	Rural	Saptari
P 7	M	26–30	Higher secondary level	Social Mobiliser	Rural	Saptari
P 8	F	41–45	Secondary level	FCHV	Rural	Saptari
P 9	M	36–40	Secondary level	Ward Chairperson	Rural	Saptari
P 10	F	21–25	Higher secondary level	Healthcare worker	Urban	Parsa
P 11	F	41–45	No formal education	Farmer	Urban	Parsa
P 12	F	21–25	No formal education	Housewife	Urban	Parsa
P 13	M	31–35	University education	Teacher	Urban	Siraha
P 14	F	41–45	No formal education	Wage based labor	Urban	Siraha
P 15	F	36–40	Secondary level	FCHV	Rural	Sarlahi
P 16	F	21–25	Secondary level	Student	Rural	Sarlahi
P 17	M	71–75	Primary level	Ward Chairperson	Rural	Sarlahi
P 18	M	31–35	Higher secondary level	Farmer	Rural	Sarlahi
P 19	M	36–40	University education	Farmer	Urban	Sarlahi
P 20	M	61–65	Primary level	Ward Chairperson	Urban	Sarlahi
P 21	M	31–35	Primary level	Tailoring	Urban	Sarlahi
P 22	F	31–35	No formal education	Farmer	Urban	Sarlahi
P 23	F	36–40	Primary level	FCHV	Urban	Sarlahi
P 24	M	46–50	Secondary level	Social Worker	Urban	Parsa
P 25	F	41–45	University education	Social Worker	Urban	Parsa
P 26	F	26–30	University education	Journalist	Urban	Dhanusha
P 27	F	46–40	University education	Social Worker	Urban	Parsa
P 28	M	61–65	University education	Retired Civil Worker	Urban	Dhanusha
P 29	M	51–55	Secondary level	Farmer	Rural	Siraha
P 30	M	36–40	University education	Ward Chairperson	Urban	Siraha
P 31	M	36–40	University education	Teacher	Urban	Siraha
P 32	M	36–40	University education	Farmer	Rural	Siraha
P 33	M	46–50	Higher secondary level	Farmer	Rural	Rautahat
P 34	M	26–30	Higher secondary level	Health worker	Urban	Rautahat
P 35	F	36–40	Secondary level	Housewife	Rural	Rautahat
P 36	M	51–55	University education	Teacher	Urban	Bara
P 37	F	36–40	University education	Human right Activist	Urban	Bara
P 38	M	26–30	Higher secondary level	Health Worker	Urban	Mohattari
P 39	M	46–50	Higher secondary level	Farmer	Urban	Mohattari
P 40	F	26–30	University education	Health worker	Urban	Bara
P 41	F	21–25	Secondary level	FCHV	Urban	Siraha

No formal education: Never attended school, Primary level: Grade (1–5), Secondary level: Grade (6–10), Higher secondary level: Grade (11–12 or equivalent), University education: Undergraduate and Postgraduate; FCHV: Female Community Health Volunteer; M:Male; F:Female

### Data collection

This study utilized semi-structured qualitative interviews to collect data. All interviews were conducted during July and August 2020. The semi-structured interviews provided flexibility to conduct the interviews by remaining focused on the research topic [31]. Due to the COVID-19 pandemic, opportunities for face-to-face interviews were limited and therefore interviews were conducted virtually using mobile phones [32]. Firstly, the interviewer made the phone calls to receive verbal consent and book an appointment for interviews. In the second step, the interviewer made another call at the agreed time to conduct an interview and ensure that participants were at ease for the interviews. Each interview lasted for about 25–30 min. The semi-structured interview guidelines intended to explore the information related to participants’ characteristics, environmental factors, health behavior, health services availability, accessibility and utilization. The interviews were conducted in *Maithili*, *Awadhi*, *Bhojpuri*, and *Nepali* languages using pretested interview guidelines (Additional file 1) by the authors DRSi (MSc), SKS (MPH), and DRSu (MSc) who are fluent in local languages. They were guided by RKS (MSc, PhD) and BA (MD, DPhil), who have experience and expertise in conducting and publishing qualitative research. The interviews were recorded on the mobile phone and the files were transferred to the password protected folder on the computer. Interviews were transcribed and translated into the English language. An independent member of the research team checked for the quality and completeness of the transcription and translation.

### Data analysis

The data were analyzed using thematic network analysis [33] in NVivo 12 Pro (QRS International, London, United Kingdom). The data analysis started after completing 23 interviews. An inductive approach to coding was undertaken to identify basic themes. Three investigators (DRSi (MSc), RKS (MSc, PhD), and LKS (MSc)) identified and reviewed the basic themes from the first five interviews to avoid lone researcher bias in qualitative research [34]. After participant-37, no new basic themes were identified, therefore indicated the saturation of the data [35]. The basic themes indicating similar issues were brought together to produce organizing themes, which enhanced the meaning and significance of the excerpts. By using thematic network analysis [33], the global themes encompassed several organizing themes to present an argument on a specific issue (Additional file 2).

### Ethical considerations

The ethical approval for this study was obtained from the ethical review board of the Nepal Health Research Council (Reference no: 2658). Participants were informed that their participation in this study was voluntary and they had the right to stop the conversation and withdraw from the study. Before commencing an interview, each participant was explained about the purpose of the study and its benefits and possible harms. Participants were encouraged to ask questions at any stage of the interview. The verbal consent from each study participant was audio-recorded and written informed consent was obtained electronically through the mobile short message services (SMS). Confidentiality and anonymity of the research participants were maintained at all stages of the research process.

## Results

Using thematic network analysis [33] the findings from this qualitative research were categorized into four interlinked global themes. The results in this section are presented under the four global themes: i) Community and stakeholders’ perceptions towards COVID-19; ii) Impact of COVID-19 and lockdown on health services delivery; iii) Community perceptions and experiences of health services during COVID-19; and, iv) COVID-19: testing, isolation and quarantine services.

### Community and stakeholders’ perceptions towards COVID-19

Most participants in this study expressed their fear and worry about the outbreak of COVID-19 due to an increasing number of cases and deaths related to COVID-19 globally and in Nepal. In villages sharing a porous border with India, community members were more scared of COVID-19 because of the increasing number of cases and deaths in the neighboring villages across the border. They were mainly concerned about the risk of an open border and mobile trade across the border, which could potentially bring diseases to their villages. Nonetheless, over the period, the fear seems to have decreased, particularly due to the low mortality rate.

*People were very afraid at the beginning as the cases were increasing rapidly. At present people's fear has been declining since the death rate is low but this doesn’t mean that we are safe in the coming days. (Participant-19, 36–40 years male, Sarlahi district)*

While few participants seemed to be complacent due to the perceptions of low mortality rate, other respondents, especially those who were at the urban hotspots seemed to be worried because of the increasing rates of infection, and associated morbidity and mortality among their acquaintances, relatives, family members, and leaders.

*Birjung city is a major entry point to Nepal from India. We are in the red zone for COVID-19. Till date, about 21 people have lost their lives in our community due to COVID-19. People are struggling to get health services. COVID-19 cases are both in hospitals and in the community. People are also staying in isolation. Many people are tested positive with COVID-19. Even community leaders and political leaders are infected here in Birjung. Local leaders including the Mayor of all municipalities in our district are infected with COVID-19. The situation is very fearful. (Participant 24, 46–50 years male, Parsa district)*

Most respondents reported that the increasing spread of COVID-19 infection among community members was mostly associated with a lack of accurate information about the disease and stigma attached to COVID-19 which may have prompted them to conceal the disease. Concealment inevitably led to a lack of early health-seeking behavior and thus transmission among the family members and neighbors has increased. Fear of discrimination and ostracization from the community were some of the major issues for those suffering from COVID-19.

*You know it (COVID-19) is contagious and at the beginning, people were not aware of how to protect from this disease. One of my relatives was infected with COVID-19, he was very afraid. He thought that if he reveals the disease he will be put into the hospital and may die without the support from his family. Later all 8 members from his family were infected, one of them being 8 months pregnant. You know why this happened; people have no adequate understanding of this disease. In the beginning, the only slogan we heard was - wash your hands and stay at home - but they did not know why? Slowly people have started to understand the meaning. Still, not all people know what this pandemic is about and what to follow to stop the transmission. People do not understand until he/she gets the disease. Ignorance is a major reason for the transmission of this disease. People have a stigma towards this disease. People have a discriminatory attitude and talk negatively about those suffering from COVID, even after they have recovered … People have created such feelings that if someone is infected with COVID, they are treated as criminals and this is putting mental pressure on such people. (Participant 25, 41–45 years female, Parsa district)*

There were clear differences between the socio-demographic characteristics of the participants when it came to following or abiding by public health preventative measures such as social distancing, wearing facemasks, and maintaining hygiene. For instance, community members from rural areas found it difficult to abide by the measures compared to the people from urban cities, with higher levels of education and high socio-economic status.

*People who are educated and handle technology, especially social media, they are getting updated information about COVID-19 every minute. They know what is happing around the world. Initially, after lockdown, people took precautionary measures but now when I go to the market, I see poor people are not following any preventive measures. I saw people who came to the hospital from rural areas were not wearing masks and not maintaining social distance while those from the urban settlement were wearing masks and were conscious about maintaining social distancing. This may be because rural people do not have access to masks, or this might be due to a lack of awareness and information about COVID. (Participant 27, 36–40 years female, Parsa district)*

### Impact of COVID-19 and lockdown on health services delivery

Most participants in this research agreed that the lockdown measures had a visible impact on all aspects of health services delivery. Although their perceptions about the level of disruptions varied, most reported that immunization, maternity services, and supply of medicine were the worst affected areas of health care delivery.

*People suffered a lot during this lockdown period. No services were available for a few months, even general health services were not available. Child and maternal health services were totally affected at the community health post. Child immunization was halted for months. Later, the local health facility started to provide immunization services, but most parents did not want to go to the health facility for their child’s immunization because of the fear of getting COVID. Older adults who were suffering from non-communicable diseases like diabetes, hypertension, had to go without medicine for several weeks and it was a very terrible situation. We could not even get iron tablets for pregnant women during the lockdown. Local private pharmacies remained closed for 3-4 months, they did not even care for any emergency situation, even if people were dying or suffering from complications of long-term illness. We had to go to the city for medicines which is far from our village. (Participant 11, 41–45 years female, Parsa district)*

The disruption in health services was echoed by the local health care workers who reflected on how the COVID-19 changed their clinical practice and the quality of services they provided to the patients. As a result of the disruption in health services, community-based FCHVs received increased consultation and were burdened to provide services to patients, although they had limited resources and capacities.

*Health services were interrupted for few months … … .For the first three months, we did not have PPE, even we did not have gloves and masks, as a result, we [health workers] stopped providing maternal and other general health services, although the health post was open … … We just provided medicine through a window by listening to their signs/symptoms. Patients were referred to primary health care centers near to our community and most of the cases were also referred to hospitals at the district headquarter which is 20 kilometers [away] from our village … … … … … (Participant 10, 21–25 years female, Parsa district)*

*We (FCHVs) were told by health workers to inform community people not to visit health facilities as health service providers would not respond to them at the health facility. We took life risks to serve people from our community during this COVID crisis though we are not trained to deal with such health issues. (Participant 1, 46–50 years female, Saptari district)*

Disruption in health services was echoed across by other stakeholders such as social workers who particularly expressed concerns around poor preparedness and arrangements in the hospital for COVID-19 patients, the reluctance of doctors to attend patients, lack of ambulance services amidst the rising cases of COVID-19, and deaths. Nonetheless, few community leaders seem to defend and asserted that health services were continuously provided.

The lack of coordination and pre-emptive preparation among the three tiers of government was recognized as one of the main reasons for disruption in health services. In addition to the poor coordination and preparedness, the spread of infection among health workers further jeopardized the capacity to respond to COVID-19 cases among the population.

*We have three levels of government but there is no adequate coordination amongst them. When COVID cases started to increase, they closed local hospitals and had no plans for COVID-19 hospital, as a result, existing health facilities struggled to meet the needs and therefore it has created serious disruptions in general health services. We had 39 health workers who were tested positive in our government hospital. After that health workers are not admitting any patients. The number of healthcare workers has started to decrease due to COVID-19 infection and this has directly affected the services in our hospital. (Participant 27, 36–40 years female, Parsa district)*

### Community perceptions and experiences of health services during COVID-19

Community members perceived that the risk of transmission of infection was high at the hospitals and therefore expressed concerns about visiting health facilities. These concerns affected people’s health-seeking behavior and they were reluctant to seek health care on time or unless absolutely necessary.

*People are very worried and are not willing to go to health facilities even if they have general health problems. They used to contact health personnel through phone calls, but they were not willing to visit any health centers due to fear of getting COVID-19 from health workers. (Participant 1, 46–50 years female, Saptari district)*

Fear of transmission was pervasive among health workers as well. Participants perceived the reluctance of health care workers in providing health services due to fear of transmission of COVID-19.

*Health workers were very afraid to provide services at the local health facility. They did not touch or examine the patient properly and did not give enough time for consultation with the patient. They just used to give medicine by maintaining the distance. (Participant 22, 31–35 years female, Sarlahi district)*

When participants had to seek health care for severe cases, the other major barriers were the availability of transportation means, particularly during the lockdown. While few were able to connect to the health care workers remotely through mobile phones, much of the health services were severely hindered by a lack of transportation services. Availability of ambulance services were also constrained during this lockdown and few participants shared their narrative on how some of them had struggled to get an ambulance service.

*The transportation facilities are hugely affected, as a result, people have problems in reaching health facilities and receiving health services in time. One of my neighbors had a leg fracture and he could not reach the hospital due to a lack of transportation services. One pregnant woman in my village had a problem during this lockdown, their families were very worried, as they could not reach the health facility due to lack of transportation. (Participant 14, 41–45 years female, Siraha district)*

Participants claimed that people had to seek private health care services as an alternative option, which was more expensive and unaffordable for many of the vulnerable population from the poor and disadvantaged communities who were worst affected and were at higher risk of COVID-19. Despite these pre-existing barriers, private hospitals were more restrictive in treating patients and often demanded COVID tests (which involved higher costs and was done only in a few designated COVID-19 hospitals/laboratories) before they could admit the patients. Although tests are justifiable and are critical before initiating the treatment to stop further spread of COVID-19, tests as a pre-requisite for health care were also perceived as a deterrent by participants.

*Private hospitals are very careless as they are not responding to support during this pandemic situation. The community has pressured the health chief of major hospitals in the city, now they have slowly started to respond to patients. Even if people are admitted they have to test for COVID first. People have perceptions that without the COVID-19 test, none of the hospitals will admit patients. So, many people are not going to hospitals even if they need health services. (Participant 24, 46–50 years male, Parsa district)*

Even if community members were able to get the test done, the quality of care remained very poor, and sometimes, family members had to visit multiple hospitals to admit patients for treatment. As a result, some of these patients lost their lives in transition.

### COVID-19: testing, isolation, and quarantine services

Participants in this research claimed that the government has not planned well for the COVID-19 pandemic, which has affected the overall health services delivery for the local population. The establishment of COVID-dedicated hospitals without allocating health services for non-COVID illnesses was particularly seen to be problematic. This meant the patients using services from those local hospitals had to seek health services from other health care facilities, which were overstretched and far from their usual health service centers.

*At the beginning of the outbreak, our district hospital, which is the only major hospital in this district, was made a dedicated isolation center for COVID-19. This has hampered all the secondary level of health care services for thousands of people who used to visit this facility as a referral center from different health posts within the districts. Now, we need to go to the nearby city general health services. (Participant 37, 36–40 years female, Bara district)*

While COVID-19 designated hospitals already affected the non-COVID health services, COVID related health services were inefficient. For instance, the availability of COVID-19 testing was limited including the high costs and long time for the test results.

*People are getting COVID-19 test reports in more than a week. The government has said people can go to a private hospital for a COVID test by paying NRS.5500 (≈55 United States Dollars) but how can general people pay this amount. (Participant 28, 61–65 years male, Dhanusha district)*

Participants reported that the isolation and quarantine services were not adequate to provide effective care for COVID-19 patients, which was additionally complicated by the politicization of the services. Specifically, political affiliation and people with power and privilege exercised the health facilities, leaving the poor and vulnerable without services.

*… at the quarantine center, we do not have a good facility. The center is overcrowded, patients are given only two meals per day and there is no separate toilet facility for males and females. People are afraid to stay at the quarantine center and even if they have symptoms, they try to hide them. If someone is close to the local leaders or affiliated with the leader’s party then he/she can have all services keeping them on high priority. For normal people, it is really very difficult to survive there. Sometimes leaders directly say that those who have not voted for them will not get these services. We feel discriminated by our local leaders based on caste and votes. Dalits and other castes (not caste of local leaders) are left behind and nobody cares about that. We are victims of these discrimination practices at quarantine and health facilities here within our own community. (Participant 35, 36–40 years female, Rautahat district)*

Testing, isolation, and quarantine services were also affected by the lack of coordination between the three tiers of governance with poor accountability and responsibilities by each one of these tiers. Task shifting and blame-game were found prominent among these authorities when discussed the responsibilities; and cited the constraints in the management of COVID-19 testing and management.

*Our three tiers of government are totally confused about their roles and responsibilities. Suppose if a person is tested positive with COVID-19, there is no clear understanding of where to seek health services. Sometimes local authorities’ say it is not their responsibility, the again provincial government says it is not their duty and at the end, patients have to lose [their health and lives] because of their conflicts of power clash and ambiguity in policies. Whose responsibility is testing and tracing, public health office says it’s not my duty, the local authority says it’s not my duty, the hospital says it’s not my duty so whose duty is this? (Participant 24, 46–50 years male, Parsa district)*

## Discussion

This study has explored community perceptions of COVID-19 and experiences related to health services utilization during the COVID-19 pandemic in Province-2 of Nepal. Using the Andersen’s model of health care utilization [23], this study found that the COVID-19 pandemic has acted as an external factor to highlight the weaknesses of the recently reformed health care system of Nepal, particularly how it is affecting the health services utilization. Health services utilization was found to be affected by characteristics of population inherent in predisposing factors (such as age, sex, education, occupation, and residence), enabling factors (availability, accessibility, and affordability of health services), and perceptions towards health services and the current pandemic (attitudes, values, knowledge, and understanding). COVID-19 has affected existing dynamics of health services utilization including its stakeholders such as changes in the behavior of health workers, the politicization of health services, and consequent adversities.

Most participants in this study expressed distress and fear towards COVID-19, not only due to the perception that COVID-19 is a highly fatal disease but also due to prejudice of uncertainty about the pandemic situation. The ardency of fear and anxiety varied based on locality, the pervasiveness of cases, and population demographics. The stigma attached to COVID-19 among the community members has triggered a well-established mechanism i.e. ‘concealment’ of the condition to avert discrimination which nonetheless can be detrimental as it can hinder early health-seeking behavior.

A previous study from 28 countries highlighted that social stigma attached to COVID-19 has led to perceived risks and fears of losing loved ones, facing intolerance, and other discriminatory acts [36]. Moreover, the continued rapid increase in the number of COVID-19 cases accompanied by an “infodemic” might have fueled anxiety and fear among the local population [10]. Community members have further seen and heard how COVID-19 patients are stigmatized at hospitals, restricted to be visited by their close family members. Such multi-faceted acts of discrimination towards COVID-19 have added to the perceived fear and stigma towards the disease [37]. Inevitably, the higher epidemiological burden of the disease, porous border with India, contributing to high movements of labor migrants, together with population vulnerability (due to high poverty, low literacy, and access to health services) has crippled health services in the South Eastern plains, particularly province-2 of Nepal. Moreover, perceptions towards health services and the knowledge, attitude, and understanding of the current pandemic have further affected the health service delivery and health care utilization.

In our study, health care providers, including health workers, were also found worried about catching COVID-19 and they were reluctant in delivering health services without adequate supplies of PPE. A recent quantitative study in Nepal found more than one-third of health care workers working in COVID-19 designated hospitals had fear and anxiety symptoms [38]. Nepal is one of the resource-poor countries and heavily relies on donors’ support for health care, which may well have affected the availability of PPE for health care workers. Globally and in Nepal, fear and anxiety among health care providers were found to be associated with the scarcity of personal protective gear and fear of transmission [38–40]. Fear about the disease among the community to some extent may play a positive role in increasing compliance with public health measures imposed by the government. However, evidence suggests “fear appeal” or “threatening communication” about the diseases is not sufficient to change preventive behavior among the public if adequate pragmatic coping strategies are insufficient [41].

Lockdown had pervasive impacts on health services, including disruptions in routine and essential health care such as immunization, maternity services, and supply chain for medicines and equipment. Our findings are also echoed by a recent study that found a reduction in institutional delivery by half and an increase in neonatal mortality by more than three times during the lockdown period [13]. Nonetheless, this study highlights how such services were disrupted in primary, secondary, and tertiary care centers of Province 2 since these centers were converted into COVID-19 dedicated hospitals.

Long-term closure of transportation services was found as one of the major hindering factors for accessing health services in both rural and urban settings. Several previous studies have also highlighted that lockdown and transportation closures have added to the extreme hardships among the general population in different parts of Nepal that ranged from not being able to fulfill their routine activities and importantly, not being able to attend the health services for non- COVID illnesses [6, 8].

The current lockdown situation has created unemployment and job scarcity, particularly for daily wage-based laborers, and this could have a direct impact on their health and wellbeing status [42]. A review study from the neighboring country India also supports these findings with an argument that the COVID-19 pandemic will push a significant number of the population below the poverty line, creating higher socioeconomic inequalities suppressing the enabling factors that will have further detrimental effects on health and health care utilization status [43]. In Nepal, the vulnerable population is composed of Dalits and marginalized population, in addition to women, children, and older adults who are already suffering from hunger, poor health, discrimination, and political powerlessness (during normal situation); these disadvantaged groups have suffered disproportionately from disasters such as earthquakes, floods and now the current pandemic [44–46].

In our study, FCHVs played a vital role in supporting community health care when the majority of health facilities were closed or disrupted. FCHVs enabled the accessibility of health services by visiting households with pregnant women or mothers with children under 5 years to collect regular updates on the health status of the mother and the child and to communicate with them about the risk of COVID-19. These findings are supported by the fact that FCHVs have been established as ‘community infrastructure’ in Nepal’s health care system, especially in rural settings for the past three decades in achieving Millennium Development Goals (MDGs) [19]. However, during the interaction with FCHVs in this study, their major grievances were around how they lacked adequate knowledge on COVID-19 to assist community members in addition to limited or no access to PPE, and lack of incentives or daily allowances. It should be considered unethical to anticipate FCHVs to carry out additional duties without being able to receive reasonable and additional benefits. It is essential for the local and national government to acknowledge the work of FCHVs and make them feel valued with appropriate incentives, including safeguarding during the COVID-19 pandemic.

Community members reported how the COVID-19 pandemic affected essential health services which had pervasive impacts on their routine and emergency health services. The scaling down of the public health services also meant that private health facilities were the only options but that were expensive and the costs were further increased during the pandemic making it increasingly unaffordable for the majority of people from a poor and disadvantaged community. This inevitably affected all populations and predisposing factors such as socioeconomic status came into play where the socio-economically poor and vulnerable population were severely affected towards the utilization of health services.

Mothers were deprived of antenatal services and had to deliver their babies at home in the absence of skilled health care workers and few of them lost their lives due to delays in reaching health facilities. This result is supported by the previous study that showed *Madheshi* ethnic groups have significantly lower attendance of maternal and child health services during the lockdown period as compared to other ethnic groups [13]. The childbirth services alone have declined by 17% among *Madheshi* communities during the lockdown period which may pose challenges in meeting the national health targets for sustainable development goals [13]. The majority of *Madheshi* ethnic groups reside in Province 2 of Nepal [16], therefore this ethnic discrepancy in the reduction of health service utilization explicitly indicates that maternal and child health services in the Province 2 have been largely disrupted during the lockdown period. The study conducted by Guttmacher Institute suggests that even a 10% decrease in the service coverage of essential maternal and newborn care during the COVID-19 lockdown period will have catastrophic consequences for the lives of mothers and their children [47].

Moreover, our study showed many patients with chronic diseases presenting fever as symptoms have struggled to receive health care services and lost their lives because several districts and province-level hospitals in the region declined to admit and treat such patients. However, some participants expressed that few patients with chronic diseases were able to communicate with their regular health care providers over the phone for advice. The global study conducted among health workers from 47 countries showed about two-thirds of the health care professionals rated that change in health services since the COVID-19 outbreak has severely impacted the health conditions and health services utilization among chronic disease patients such as the person with chronic obstructive pulmonary disease, hypertension and diabetes [48]. The study has also highlighted the increase in virtual communication with their patients during this crisis [48]. In this context, our study could bring the attention of health care providers to assimilate the current approach of health services delivery with the future possibility of genuine telemedicine interventions particularly for chronic disease patients who need regular follow-up and advice. The use of virtual technology could act as enabling factors for health care utilization by increasing the availability and accessibility of health services, especially for people from rural and remote areas, which will also make it more affordable and less time constraints.

The participants in our study revealed that the concerned authorities have failed to manage effective provision for testing, isolation, and quarantine services, although these services are seen at the heart of effective public health responses to COVID-19 [49]. Although the government of Nepal has built quarantine facilities, these are overwhelmed by a large number of migrant workers returning home from the open border of India [50]. In addition, there are no updated records of people’s movements at the border or across different places within the country. Many migrant workers endured a long-distance walking from India, who may have evaded the quarantine services and were perceived to be fueling the community transmission [51]. Participants in our study reported that many people escaped from hospitals and quarantine centers. The reason for this is the shortfall of public trust towards the public health system of Nepal. Also, most of the quarantine and isolation centers lacked basic infrastructures, with some sporadic stories of poor security, gender harassment, and even rape, which may have demotivated the migrant returnees to stay [37]. The government had managed COVID-19 test facilities in limited public hospitals. This shows a lack of effective preparedness, planning, and coordination for delivering testing and quarantine services when there is an increasing number of COVID cases [37, 50]. In addition, the inadequate management of isolation and quarantine services are found greatly influenced by favoritism and discrimination based on political affiliation in Nepal. Furthermore, people are experiencing delays in receiving the test report, sometimes even more than 2 weeks. A recent study has highlighted the gaps in coordination and lack of clear roles and responsibilities between three tiers of newly reformed government structures in responding to COVID-19 [8]. Though the government has also arranged a COVID-19 testing facility at private health centers, it is too expensive that cost NRS 5500 (~ USD 55.0), and is unaffordable for the general population where 32% of the population live with earnings between USD 1.90 to USD 3.20 per day [52]. Overall, the health care utilization among the population during COVID-19 has been affected by complex of factors including attitude and perceptions towards health service providers, knowledge and understanding of the COVID-19 pandemic, and the availability, accessibility, and affordability of health services.

### Strengths and limitations

This study was conducted in province-2 of Nepal, which has high population density, with relatively poor health status indicators, and shares a porous border with India in all its 8 districts, thus the findings of this study may not resonate with other parts of Nepal, especially mountain and hill regions. This research is conducted during the COVID-19 pandemic when the health care needs were high, therefore the findings of this study need to be associated with the context of COVID-19 and it is likely that the community perceptions towards health services utilization may vary with the relaxation of the lockdown and after the pandemic. However, the results of our study can be utilized for future policy and program planning to deliver effective health services during disasters and health emergencies in Nepal.

## Conclusions

The study concludes that public fear and anxiety of COVID-19, transportation disruptions, unavailability of medicines and health services at local health facilities, stigma related to COVID-19, poor management of quarantine, isolation and testing facilities of COVID-19, demotivation among health care providers, and limited involvement of private health care sectors were major barriers in health services utilization during COVID-19 pandemic in Province-2 of Nepal. In addition, essential health services such as maternal and child health services, chronic diseases treatment facilities were severely affected in both rural and urban settings. In addition to COVID-dedicated health services, essential health services should receive a continuous priority to optimize the uptake of health care utilization among the local population.

## Supplementary Information

**Additional file 1.** Interview guide (English version).

**Additional file 2.** Thematic network analysis framework (from codes to global themes).

## Data Availability

The interview transcripts generated and analyzed during this study are not publicly available to maintain the confidentiality of the participants but are available from the corresponding author on reasonable request.

## Abbreviations

COVID-19: Coronavirus Disease-2019
FCHVs: Female Community Health Volunteers
PCR: Polymerase Chain Reaction
PPE: Personal Protective Equipments
SMS: Short Message Services
USD: United States Dollar
