# Supplementary material for: Impact of COVID-19 on health services utilization in Province-2 of Nepal: a qualitative study among community members and stakeholders
Source: BMC Health Serv Res. 2021 Feb 24;21:174. doi: 10.1186/s12913-021-06176-y (PMC7903406; doi:10.1186/s12913-021-06176-y)
Supplement: Supplementary file 1 — Additional file 1. Interview guide (English version). [file 12913_2021_6176_MOESM1_ESM.docx]

**Additional file 1. Interview guide (English version)**

**INTERVIEW guide**

| **Section A: Socio-demographic data** | |
| --- | --- |
| 1. Please introduce yourself?  2. How old are you?  3. What is your education level?  4. What is your occupation?  5. Sex of the participant: | |
| **Participants residence details:**  6. Name of district :  7. Name of Rural/Urban municipality:  8. Ward number: | |
| 9. Date of Interview: | Date Month Year |
| 10. Name of Interviewer: | …………………………………………… |

**Section B: Guiding questions related to COVID-19 and health services utilization during the COVID-19 pandemic.**

- In your opinion, what is the situation of COVID-19 in your community and how have you perceived the risk of COVID-19? Please explain.
- Are there any COVID-19 cases in your community? If yes, where does he/she is receiving treatment? What is the provision of receiving COVID-19 related health services in your community? Please explain.
- In your observation or experiences how you or people in your community are utilizing non-COVID-related health services during this pandemic?
- Could you please share your experiences or efforts in delivering health services during the pandemic? (only ask to health workers, female community health volunteers, and local elected authorities)
- How people in your community are accessing health services during the lock-down period? In your observation, what kind of people are most affected in accessing health services during this pandemic?
- In your experiences or observation, what are the effects on the general health services availability during this lockdown period? Can you please also provide some examples?
- How the mothers, children, and elderly people are receiving health services other than COVID-19 during this pandemic?
  - -Probing to explore more with some examples.
- How the local health facility is providing general health services to community people during this lock-down period?
- In your observation or experiences, how the different levels of governments are responding to this pandemic particularly in providing health services to community people?
- Please feel free to share, if you have any suggestions or anything you would like to say or you think you have missed during our conversation?
