# Supplementary material for: Impact of COVID-19 on health services utilization in Province-2 of Nepal: a qualitative study among community members and stakeholders
Source: BMC Health Serv Res. 2021 Feb 24;21:174. doi: 10.1186/s12913-021-06176-y (PMC7903406; doi:10.1186/s12913-021-06176-y)
Supplement: Supplementary file 2 — Additional file 2. Thematic network analysis framework (from codes to global themes). [file 12913_2021_6176_MOESM2_ESM.docx]

**Additional file 2: Thematic network analysis framework of the study (from codes to global themes)**

| **Codes** | **Basic themes** | **Organizing themes** | **Global themes** |
| --- | --- | --- | --- |
| - Maternal health - Child health - Immunization - Fever examination - Anxiety - Fear - Pandemic - Patient number - Transportation disruptions - Childbirth at home - Maternal deaths - Private health facility - Affordability of services - Deprivation of services - Practices at health facilities - Awareness - Alternative health care - Shortage of essential medicines - Shortage of PPE - Community perceptions - Quarantine facility - Conflicts at quarantine - Mental health problems - COVID-19 test - COVID-19 tracing - Ignorance - Isolation centers - Absenteeism of health workers - Lack of resources - Health worker incentives - FCHVs support - management - Emergency health plan - Poor response - Sanitation and hygiene - Health awareness - Sanitation at health facility - Migrant returnees - Response towards migrant returnees - Mental problems - stigma - Open boarder - Migrant’s returnees - COVID-19 transmission risks from migrants’ returnees - Community expectations | 1. Anxiety and fear among community 2. Fear and anxiety among health workers 3. Community perception towards health worker 4. Maternal and child health services utilization 5. Child immunization services 6. Health services at private health facilities 7. Availability of general health services 8. Challenges in services accessibility 9. Disruption of essential medicines and medical supplies 10. Community perception towards health facilities 11. Information on COVID-19 12. Private health sectors involvement in COVID-19 health services 13. Closures of health facilities 14. Roles of female community health volunteers 15. Availability of alternative health services 16. Decrease in numbers of patients at local health facility 17. Home based self-care 18. Obstruction of transportation services 19. Female community health volunteers support during COVID-19 crisis 20. COVID-19 screening and testing 21. Quarantine facilities management 22. Stigmatization towards COVID-19 infection 23. Open border crossing 24. Health risks from migrant returnees 25. Behaviour towards migrant returnees 26. Discrimination in services at quarantine and isolation centers | 1. Anxiety and fear among community population, including health workers, due to COVID-19 pandemic 2. Disruptions of transportation including ambulance services 3. Lack of information, stigma and ignorance 4. Resources and hygiene and sanitation at health facilities 5. Health worker reluctance in providing health care both at public and private health facilities 6. Government response – health service disruptions 7. FCHVs involvement in COVID-19 services 8. Community perceptions towards health workers and health facility 9. Increase in COVID-19 cases and deaths 10. Awareness on COVID-19 among community 11. Unpreparedness, lack of planning and management 12. Poor monitoring at open border crossing between India and Nepal 13. Poor quarantine facilities and tracing for COVID-19 14. Delayed in testing for COVID-19 15. Discriminatory behaviors from local authorities at quarantine and isolations centers 16. Perceived health risks and behavior towards migrants’ returnees | 1. Community perceptions towards COVID-19 2. Impact of lockdown on health services delivery 3. Community perceptions and experiences of health services during COVID-19 4. COVID-19: testing, isolation and quarantine services |
